# A Study on the Shrinkage and Compressive Strength of GGBFS and Metakaolin Base Geopolymer under Different NaOH Concentrations

**DOI:** 10.3390/ma17051181

**Published:** 2024-03-03

**Authors:** Yen-Chun Chen, Wei-Hao Lee, Ta-Wui Cheng, Yeou-Fong Li

**Affiliations:** 1 Institute of Mineral Resources Engineering, National Taipei University of Technology, Taipei 10608, Taiwan; t107799001@ntut.edu.tw (Y.-C.C.); twcheng@ntut.edu.tw (T.-W.C.); 2 Department of Civil Engineering, National Taipei University of Technology, Taipei 10608, Taiwan

**Keywords:** geopolymer, ground granulated blast furnace slag, metakaolin, shrinkage, compressive strength

## Abstract

Geopolymers (GPs) are gaining prominence due to their low carbon emissions and sustainable attributes. However, one challenge with GPs, particularly those made with ground granulated blast furnace slag (GGBFS), is their significant shrinkage during the geopolymerization process, limiting its practical applicability. This study focuses on how the substitution ratio of metakaolin (MK) and the concentration of sodium hydroxide (NaOH) in the activator can influence the shrinkage and strength of a GGBFS-based GP. The experimental approach employed a 3 × 3 parameter matrix, which varied MK substitution ratios (0%, 50%, and 100%) and adjusted the NaOH concentration (6 M, 10 M, and 14 M). The results revealed that increasing MK substitution, particularly with 6 M NaOH activation, reduced the GP shrinkage but also diminished compressive strength, requiring higher NaOH concentrations for strength improvement. Statistical tools, including analysis of variance (ANOVA) and second-order response surface methodology (RSM), were employed for analysis. ANOVA results indicated the significant impacts of both the MK content and NaOH concentration on compressive strength, with no observable interaction. However, the shrinkage exhibited a clear interaction between MK content and NaOH concentration. The RSM model accurately predicted compressive strength and shrinkage, demonstrating a high predictive accuracy, for which the coefficients of determination (R^2^) were 0.99 and 0.98, respectively. The model provides a reliable method for determining the necessary compressive strength and shrinkage for GGBFS-based GP based on MK substitution and NaOH concentration. Within the optimization range, the RSM model compared with experimental results showed a 6.04% error in compressive strength and 0.77% error in shrinkage for one interpolated parameter set. This study establishes an optimized parameter range ensuring a GP performance that is comparable to or surpassing OPC, with a parameter set achieving a compressive strength of 34.9 MPa and shrinkage of 0.287% at 28 days.

## 1. Introduction

A geopolymer (GP) is a type of alkali-activated material (AAM) crafted from fine powders rich in silicon and aluminum. These powders are dissolved in alkali solutions, forming a sodium alumino-silicate hydrate (N-A-S-H) gel [1,2], which gives GPs their unique properties. The structure of a GP is akin to zeolite, existing in amorphous or semi-crystalline forms [3,4]. When calcium-rich materials like ground granulated blast furnace slag (GGBFS), CaO, or Ca(OH)_2_ are incorporated, a calcium (alumino-)silicate hydrate (C-S-H) gel forms, enhancing the mechanical properties and accelerating hardening [5,6,7,8]. This has led to some debate in academic circles, with certain studies suggesting that high-calcium-containing raw materials produce an AAM rather than a traditional GP, which is an alkali-activated material containing silicon and aluminum without calcium [9,10].

For practical applications, GP is often mixed with calcium sources from industrial by-products such as GGBFS, class-C fly ash (FA), waste ceramic powder, and oyster shell powder. The purpose is to reuse these by-products or waste from construction, industry, agriculture, and breeding as building materials to avoid an excessive accumulation and impact on the environment. This blending blurs the lines between GPs and AAMs [11,12,13]. Compared to ordinary Portland cement (OPC), GP variants like GGBFS and GGBFS/FA-based GPs not only demonstrate higher compressive strength but also significantly reduce carbon emissions—between 50 to 90% [14,15]. It is worth noting that the evaluation conditions and carbon emissions factors varied in each case and country [16]. Consequently, a GP is regarded as a low-carbon-emission material with the potential to replace OPC [17].

While GPs exhibit a higher compressive strength compared to OPC, their practical application is limited due to excessive shrinkage issues [18,19,20,21,22]. This shrinkage is primarily attributed to the consumption of nano-pore water in the GP [23]. In particular, a GGBFS-based GP activated by sodium silicate and sodium hydroxide can experience shrinkage up to three and six times greater than that of OPC, respectably. Although the activation of a GGBFS-based GP using sodium carbonate can have a similar shrinkage to that of OPC, the compressive strength will be too low [24]. Alternatives like FA and MK-based GPs offer improved volume stability compared to GGBFS-based GPs but tend to have lower strength [25]. Adding silica fume (SF) to FA-based GPs can enhance strength, yet it may also increase shrinkage [26,27,28]. Furthermore, FA’s composition can vary significantly depending on the coal source and power station types [29], leading to varying properties in GPs. Consequently, a blend of FA, GGBFS, MK, and SF has been researched extensively to strike a balance between strength and shrinkage in GP formulations [20,21,22,23,24,25,26,27,28,29,30,31].

MK as a raw material for GPs demonstrates superior volume stability compared to GGBFS-based GPs [32]. MK, similar in properties to FA and GGBFS, possesses cementitious properties and is commonly used in OPC concrete [33,34]. It is derived from calcining kaolin clay at temperatures between 600 to 850 °C for durations ranging from 1 to 12 h, depending on the kaolin’s chemical composition [35,36]. This calcination process eradicates the hydroxyl groups in the kaolin, transforming it into a more reactive, amorphous MK, ideal for effective geopolymerization [37,38,39]. Additionally, an MK-based GP is particularly suitable for repair applications, given its mechanical properties closely resembling those of OPC and its superior tensile strength [40,41,42].

Acknowledging MK’s more consistent source and composition compared to FA, this study focuses on substituting GGBFS with varying proportions of MK in a GP. It also involves adjusting the concentration of NaOH in the alkaline activator to examine the resulting changes in compressive strength and shrinkage. Given the multitude of factors influencing GPs, a comprehensive statistical analysis is utilized, including regression models and analysis of variance (ANOVA). These analytical methods are employed to assess the impacts of different parameters on GPs and to identify the optimal combinations [43,44,45,46]. The research methodology incorporates a two-variable, three-level experimental design, with the findings subjected to ANOVA and regression modeling.

## 2. Material

This study aimed to enhance the shrinkage properties of GPs by incorporating MK into a GGBFS-based GP formulation. To achieve this, a blend of GGBFS and MK was activated using a sodium-based alkaline activator. This activator comprised sodium hydroxide (NaOH), sodium silicate (Na_2_SiO_3_), and sodium aluminate (NaAlO_2_). In order to focus solely on the effects of the binder and activator, no aggregates were added to the GP mix, ensuring a more controlled study of the material’s intrinsic properties.

### 2.1. Binder

The GGBFS powder used in this research was sourced from CHC Resources Corp. (Kaohsiung City, Taiwan), Taiwan. It had a mean particle size (D_50_) of 12.33 μm, a specific surface area of 4000 cm^2^/g, and a specific gravity of 2.9. The MK, chosen for its fine particle size and chemical composition, was BURGESS No. 30 (Burgess Pigment Company, Sandersville, GA, USA). It featured an average particle size of 1.4 μm and a specific gravity of 2.63. The chemical compositions of both GGBFS and MK were meticulously analyzed using X-ray fluorescence (XRF), with the results detailed in Table 1.

### 2.2. Alkaline Activator

For the alkaline activation process, the study employed a sodium-based activator. This activator was prepared by blending tap water with NaOH, Na_2_SiO_3_, and NaAlO_2_. The concentration of the NaOH was varied among 6 M, 10 M, and 14 M, determined by the ratio of NaOH to tap water. Furthermore, the molar ratios of SiO_2_/Na_2_O and Al_2_O_3_/SiO_2_ in the activator were maintained at 1.28 and 0.02, respectively, calculated based on their total content in the mixture. These specific concentrations and ratios were critical for understanding the activation process and the resulting properties of the GP.

### 2.3. Ordinary Portland Cement

This study used Portland Type I cement (Taiwan Cement Corp., Taipei City, Taiwan) as a benchmark for evaluating GP specimens. The length change ratio of the OPC specimen at 28 days was established as a standard for assessing the adequacy of the GP specimens. Concurrently, the criterion for compressive strength at 28 days was set at a minimum of 28 MPa, in accordance with the specifications for rapid-hardening cementitious materials outlined in ASTM C928 [47].

## 3. Experiment

The test method, parameters, and preparation of the specimen are described as follows.

### 3.1. Test Method

This research adhered to the ASTM C109/C109M-20 [48] standard for compressive strength testing (specimen size is 50 mm × 50 mm × 50 mm), which necessitates the slurry’s fluidity to be within 110 ± 5%. Consequently, adjustments in the activator-to-binder ratio (A/B) were required for all specimens, ensuring compliance with the specified fluidity criteria, as verified by fluidity tests in accordance with ASTM C230/C230M-20 [49]. The length change ratio measurements and calculations were conducted following the ASTM C157-75 [50] standard (specimen size is 25 mm × 25 mm × 285 mm). Importantly, the preparation of the specimens for length change measurements maintained the same A/B ratio as those used for compressive strength testing. After a 24-h setting period, all specimens were demolded. The initial length of the length change rate specimens was recorded immediately post-demolding, then these specimens were placed in a controlled curing chamber. Length measurements for these specimens were taken at various ages, while the compressive strength tests were conducted at 28 days.

### 3.2. Experimental Design Parameter

The experimental design incorporated two variables across three levels to assess the impact of MK substitution and NaOH concentration. This resulted in nine distinct parameter sets, as detailed in Table 2. For each parameter, a minimum of three specimens were prepared to conduct both compressive strength and shrinkage tests. The A/B ratio for each parameter was determined based on achieving the targeted fluidity range of 110 ± 5%, ascertained through fluidity tests. Additionally, an OPC specimen was included for comparative analysis.

### 3.3. Preparation of Specimen

The preparation process involved mixing MK, GGBFS, and the activator for 2 to 5 min to achieve a homogeneous slurry. This slurry was then poured into molds and sealed, followed by a resting period in a room-temperature environment for 24 h. After demolding, the specimens were transferred to the curing chamber with the specified conditions of 52 ± 3% humidity and 23.2 ± 2 °C temperature.

### 3.4. ANOVA

In this study, F-tests were employed to analyze the data, focusing on comparing variances across multiple groups. This approach was chosen to ascertain the significance of the NaOH concentration and MK content in alkaline activators as critical influencing factors. The essence of the F-test involves contrasting the ratio of variation between groups against the variation within groups. This comparison aims to determine if the variation observed between groups significantly exceeds what might be expected from random fluctuations. A significant result is indicated either when the F-value surpasses the critical value, or when the *p*-value falls below the established significance level (α). In such cases, the null hypothesis, which posits no difference in population variation among the groups, is rejected. This suggests that the variation in at least one group is distinct from others. When significant differences between groups are identified, additional post-hoc analyses are conducted. These subsequent comparisons are crucial for pinpointing specific differences among the groups.

### 3.5. Second-Order Response Surface Methodology (RSM)

The second-order response surface model was used to create a predictive mathematical model. This model aimed to draw the curvature response surface of the compressive strength and length change ratio at 28 days based on the NaOH concentration and MK content to identify the qualification range. The model is established using the following formula:

(1) $$y=\beta_{0}+\beta_{1}x_{1}+\beta_{2}x_{2}+\beta_{3}x_{1}^{2}+\beta_{4}x_{2}^{2}+\beta_{5}x_{1}x_{2}$$

where $x_{1}$ is a variable of MK content, which is 0, 50 (wt.%), and 100 (wt.%) in this study; $x_{2}$ is a variable of NaOH concentration of the alkaline activator, which is 6 (M), 10 (M), and 14 (M) in this study; and $\beta_{i}$, i=0–5 are coefficients.

The equation can be expressed in matrix form as:

(2) Y=Xβ

where,

(3a) $$\beta ={\left[\begin{array}{c} \beta_{0}\\ \vdots \\ \beta_{5} \end{array}\right]}_{6\times 1}$$

(3b) $$X={\left[\begin{array}{cccccc} 1 & x_{11} & x_{21} & x_{11}^{2} & x_{21}^{2} & x_{11}x_{21}\\ & & & \vdots & & \\ 1 & x_{1n} & x_{2n} & x_{1n}^{2} & x_{2n}^{2} & x_{1n}x_{2n} \end{array}\right]}_{n\times 6}$$

(3c) $$Y={\left[\begin{array}{c} y_{1}\\ \vdots \\ y_{n} \end{array}\right]}_{n\times 1}$$

In the above equation, *n* is the number of the experimental parameters and the number of observations obtained for the corresponding experimental parameters; in this study *n* is 9. Note that n needs to be at least 6 or above to solve for 6 coefficients.

In this representation, Y is the matrix of observed responses, X is the matrix containing the values of the predictors for each observation, and β is the matrix of coefficients to be estimated. To find the coefficients, the least squares method is used, and the solution is given by:

(4) $$\beta =X^{-1}Y$$

This method aims to minimize the sum of squared errors between the observed responses and the predicted values of the model.

## 4. Results and Discussion

In this study, nine GP specimens with varying parameters and one OPC specimen were tested for comparative purposes. The water–cement ratio is 0.28 for the OPC specimen. Table 3 lists the results of the compressive strength and length change ratio at 28 days for these specimens.

### 4.1. Activator to Binder Ratio

**Figure 1:**
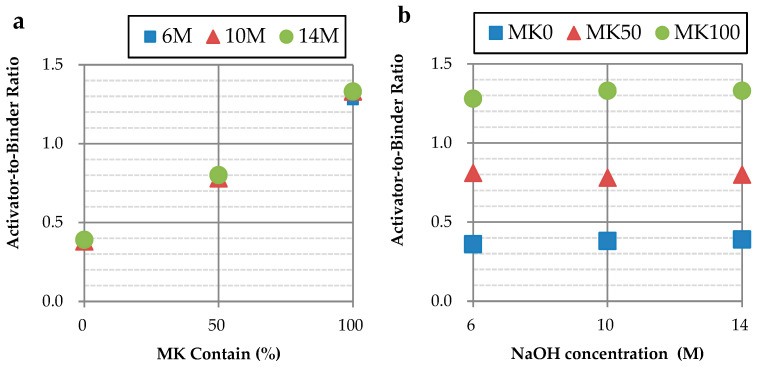
Activator-to-binder ratio of different parameters: (**a**) comparison of different concentrations; (**b**) comparison of different MK contents.

The activator-to-binder ratio (A/B) for each specimen, as presented in Table 3, was determined using a flow table test. To maintain the GP slurry’s fluidity at 110 ± 5%, an increased amount of activator was required for slurries with a higher MK content, as illustrated in Figure 1a. Additionally, it was observed that as the concentration of NaOH increased from 6 M to 14 M, the amount of required activator slightly elevated, depicted in Figure 1b.

### 4.2. Compressive Strength

**Figure 2:**
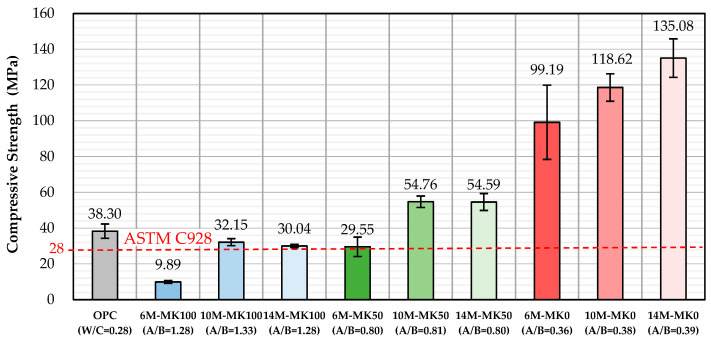
Average compressive strength of specimens at 28 days.

The compressive strength data at 28 days are detailed in Table 3 and visually represented in Figure 2. A notable trend emerged where an increase in MK content led to a decrease in the compressive strength of GP specimens. Conversely, elevating the NaOH concentration resulted in an increase in compressive strength. However, for GP specimens with over 50% MK, increasing the NaOH concentration from 10 M to 14 M did not significantly affect the compressive strength. It is worth noting that only the specimen containing 100% MK and made by 6 M activator is less than the 28 MPa specified by ASTM C928, showing that the GP generally has excellent mechanical properties. Interestingly, GP specimens either devoid of MK or containing 50% MK with NaOH concentrations above 10 M showed compressive strengths surpassing those of OPC specimens.

### 4.3. Length Change Ratio

**Figure 3:**
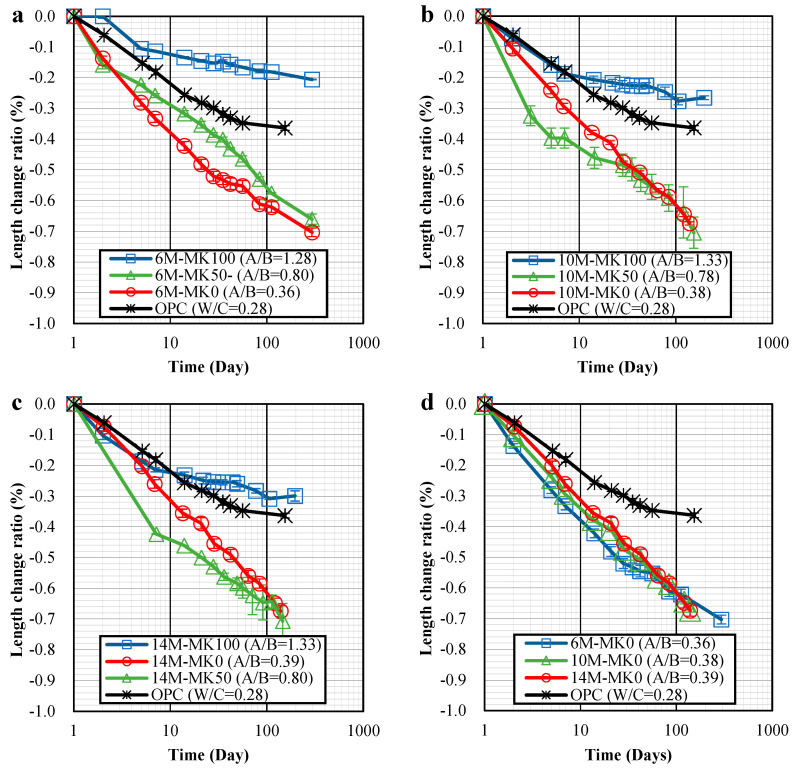

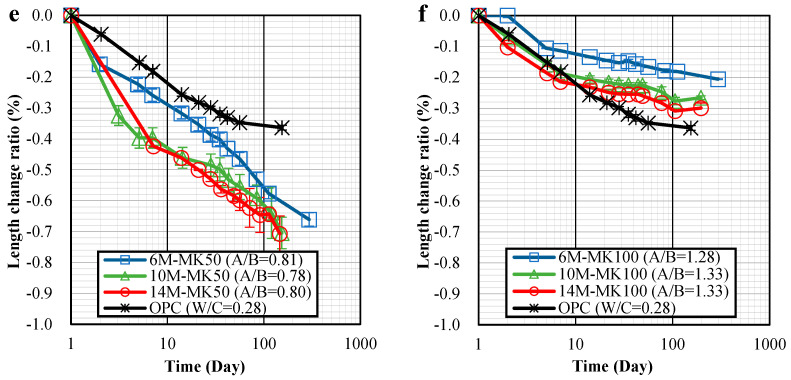
Length change ratio-time relationships of specimens: (**a**) 6 M; (**b**) 10 M; (**c**) 14 M; (**d**) MK0; (**e**) MK50; (**f**) MK100.

The length change ratios at 28 days are recorded in Table 3, while Figure 3 displays the age-related length change ratios. A key observation was that GP specimens with 100% MK content exhibited significantly less shrinkage compared to those with 0% and 50% MK. Furthermore, GP specimens with 100% MK content showed lower shrinkage ratios than the OPC specimens.

In cases where GP specimens contained 50% MK and a NaOH concentration of 6 M, a notable reduction in shrinkage was observed, as shown in Figure 3a. However, increasing the NaOH concentration to 10 M and 14 M in these specimens led to increased shrinkage at the initial stages, but their shrinkages at 28 and 100 days were comparable to those with 0% MK content, as indicated in Figure 3b,c.

It is important to note that variations in NaOH concentration had a relatively minor impact on shrinkage. For specimens with 0% MK, there was only a slight increase in 28-day shrinkage when the NaOH concentration was raised from 6 M to 14 M, as seen in Figure 3d. In contrast, for specimens with 50% and 100% MK, increasing the NaOH concentration from 6 M to 10 M and 14 M led to a noticeable increase in shrinkage, as shown in Figure 3e,f.

In conclusion, the study found that a higher MK content effectively reduced shrinkage, particularly in GP specimens with 100% MK, which exhibited lower shrinkage than OPC specimens. While reducing NaOH concentration did contribute to shrinkage reduction, its impact was less pronounced than that of MK content.

## 5. Statistical Analysis

The experimental data were analyzed through ANOVA and a regression model to make predictions. The results are as follows.

### 5.1. ANOVA

The NaOH concentration and MK content were two main factors to analyze in this study. Each has three levels, including 6 M, 10 M, and 14 M for NaOH, and 0%, 50%, and 100% for MK. This resulted in nine distinct experimental conditions, evaluated for both the compressive strength and length change ratio. To determine the primary factors influencing these response variables, a two-way ANOVA was conducted. Prior to analysis, we ensured adherence to the assumptions of normality and homogeneity of variances. The hypotheses were structured such that the null hypothesis posited equal means across groups, while the alternative hypotheses suggested notable differences. Specifically, the ANOVA for compressive strength at 28 days, detailed in Table 4, utilized a two-way repeated measures design with a significance level (α) of 5%. This method involved calculating variances within and between groups, and the total variance, along with determining the sum of squares, degrees of freedom (DoF), and mean square for each type of variance. The F-value, derived from the ratio of the mean square between groups to the mean square within groups, was compared against a critical value from an F-distribution table to gauge statistical significance. Additionally, the *p*-value associated with the F-value was examined; this value indicates the likelihood of observing such extreme results if the null hypothesis was true. The analysis showed that the interaction *p*-value between MK content and NaOH concentration exceeded 5%, suggesting no significant interaction between these variables. However, the individual *p*-values for both MK content and NaOH concentration were below 5%, confirming their significance as factors affecting compressive strength. Notably, MK content had a more pronounced impact than NaOH concentration.

Similarly, the ANOVA for the length change ratio at 28 days is presented in Table 5, and followed the same methodology as that for compressive strength. The results indicated a significant interaction between the MK content and NaOH concentration, as their *p*-values did not exceed 5%. This necessitated further analysis of each factor’s individual impact, conducted through one-way ANOVA.

The one-way ANOVA, examining six combinations for each factor, is summarized in Table 6. With a modified significance level set at 8.3 × 10^−3^, the findings showed that NaOH concentration significantly influenced the MK contents of 0% and 100%, but not 50%. Additionally, the length change ratio at 28 days was more sensitive to NaOH concentration changes for 100% MK content than for 0% MK content. In contrast, MK content had a significant impact on all tested NaOH concentrations (6 M, 10 M, and 14 M), with the 6 M concentration being the most affected by changes in MK content, followed by 10 M.

### 5.2. Second-Order RSM Model

**Figure 4:**
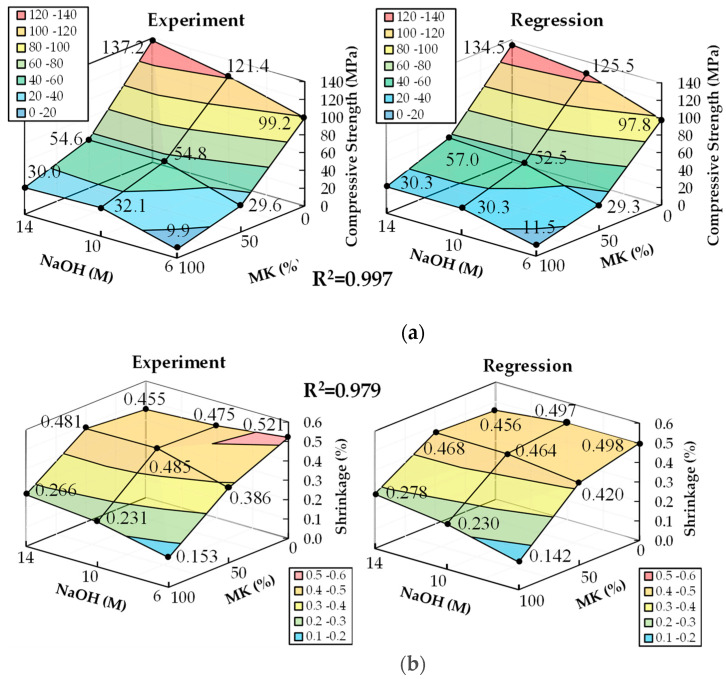
Response surfaces of RSM model and experimental results: (**a**) the average compressive strength at 28 days; (**b**) the average shrinkage at 28 days.

The coefficients β of the second-order RSM model, for the compressive strength and length change ratio at 28 days, were computed by substituting the data in Table 3 into Equations, with (3b) and (3c) as X and Y matrices, respectively. Furthermore, the coefficients β were calculated from Equation (4) and listed in Table 7. The response surface predicted from the second-order RSM model, along with the experimental results, are depicted in Figure 4. The coefficient of determination (*R*^2^) for both the compressive strength and length change ratio were 0.997 and 0.979, respectively, indicating a high degree of correlation with the experimental values.

**Figure 5:**
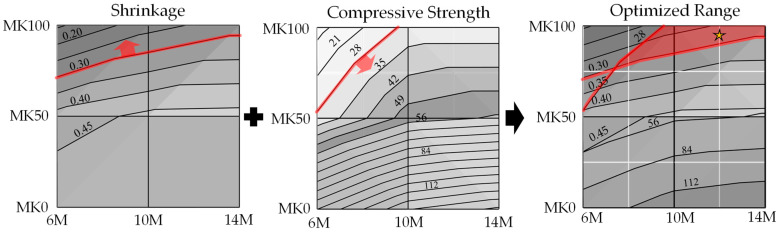
The compressive strength and shrinkage at 28 days, as calculated by the regression model, exceed 21 MPa while remaining under 0.3%. The yellow star was the chosen interpolated specimen had 95% MK content and 12 M NaOH concentration.

Figure 5 illustrates the optimal parameter range by overlaying the response surface projections for both compressive strength and shrinkage. Within this range, the compressive strength at 28 days is expected to exceed 28 MPa [47], and the shrinkage of OPC at 28 days is projected to be less than 0.3%. An interpolation experiment was conducted in this optimal range to verify the accuracy of the regression model. The chosen interpolated specimen had 95% MK content and 12 M NaOH concentration, and it is marked as a star in Figure 5. The results, as shown in Table 8, displayed an error of 6.04% for compressive strength and 0.77% for shrinkage, thereby confirming the substantial accuracy of the regression model.

## 6. Conclusions

This study conducted experimental and statistical analyses with two factors and three levels in a total of nine parameters, forming a 3 × 3 parameter matrix, which varied GGBFS/MK ratios and adjusted the NaOH concentration of a GGBFS- and metakaolin-based geopolymer. Several conclusions are listed below.

1. Higher MK content in the GP slurry necessitates an increased amount of activator to maintain fluidity. The change in the activator concentration has a less significant effect on the fluidity of the slurry.
2. GP specimens with higher MK content will reduce shrinkage, particularly when the binder contains 100% MK (MK-based GP), but this is accompanied by a decrease in compressive strength. The compressive strength of GP specimens containing over 50% GGBFS was improved by increasing the NaOH concentration. GP specimens without MK or containing 50% MK with NaOH concentrations above 10 M showed compressive strengths surpassing those of OPC specimens.
3. GP specimens with 100% MK content demonstrated significantly lower shrinkage compared to those with 0% and 50% MK content, as well as compared to OPC specimens. GP specimens with contents of 0% and 50% GGBFS exhibited long-term shrinkage after 28 days.
4. The RSM model used in the study demonstrated high accuracy, with errors of 6.04% for compressive strength and 0.77% for shrinkage, affirming its reliability in predicting the properties of GPs based on MK content and NaOH concentration.
5. The ANOVA revealed that both the MK content and NaOH concentrations are key factors affecting compressive strength, yet they exhibit no significant interaction. However, regarding shrinkage, a notable interaction between the MK content and NaOH concentration was observed, and was particularly pronounced, except for at a 50% MK content level, where their combined effect on shrinkage became significantly evident.
6. This study optimizes GP parameters to obtain the qualified compressive strength and shrinkage characteristics within the optimized range of a GGBFS/MK ratio and NaOH concentration, as shown in Figure 5. Through an interpolation experiment, the compressive strength and shrinkage of the chosen interpolated specimen are 34.9 MPa and 0.287%, respectively; those values are similar to the compressive strength and shrinkage of OPC with 38.3 MPa and 0.298%, respectively.

## Figures and Tables

**Table 1:** The chemical composition of the GGBFS and MK.

Composition	SiO_2_	Al_2_O_3_	CaO	MgO	Fe_2_O_3_	TiO_2_	Others
GGBFS (%)	32.43	9.96	47.32	5.16	0.69	0.83	3.61
MK (%)	58.15	38.34	0.19	0.00	0.90	1.66	0.76

**Table 2:** The experimental design parameters for different specimens.

Specimen	Binder		Alkaline Activator		
	MK (wt.%)	GGBFS (wt.%)	NaOH (M)	SiO_2_/Na_2_O (wt.%)	Al_2_O_3_/SiO_2_ (wt.%)
6 M-MK0	0	100	6	128	2
6 M-MK50	50	50	6		
6 M-MK100	100	0	6		
10 M-MK0	0	100	10		
10 M-MK50	50	50	10		
10 M-MK100	100	0	10		
14 M-MK0	0	100	14		
14 M-MK50	50	50	14		
14 M-MK100	100	0	14		

**Table 3:** The compressive strength and length change ratio of specimens at 28 days.

Specimen	Activator/Binder Ratio	Compressive Strength (MPa)					Length Change Ratio (%)			
		1	2	3	4	Average	1	2	3	Average
6 M-MK0	0.36	81.8	93.6	99.2	122.2	99.2	−0.539	−0.512	−0.51	−0.520
6 M-MK50	0.81	22.9	29.6	29.7	36.1	29.6	−0.408	−0.384	−0.367	−0.386
6 M-MK100	1.28	9.2	9.5	10.3	10.6	9.9	−0.156	−0.152	−0.151	−0.153
10 M-MK0	0.38	110.3	116.0	119.5	128.6	118.6	−0.478	−0.478	−0.471	−0.476
10 M-MK50	0.78	52.3	53.2	54.1	59.4	54.8	−0.514	−0.496	−0.444	−0.485
10 M-MK100	1.33	30.3	31.2	32.4	34.1	32.2	−0.248	−0.231	−0.213	−0.231
14 M-MK0	0.39	125.0	128.7	137.5	149.2	135.1	−0.472	−0.452	−0.441	−0.455
14 M-MK50	0.80	51.5	51.9	53.4	61.5	54.6	−0.535	−0.523	−0.385	−0.481
14 M-MK100	1.33	29.0	30.3	30.4	30.7	30.0	−0.271	−0.269	−0.259	−0.266
OPC	-	34.2	38.3	38.5	42.2	38.3	−0.302	−0.297	−0.295	−0.298

**Table 4:** Two-way ANOVA of the compressive strength at 28 days.

Source of Variations	Sum of Squares	DoF	Mean Square	F-Value	*p*-Value	Critical Value
MK Contain (%)	57,427.14	2	28,713.57	490.36	6.00 × 10^−22^	3.35
NaOH (M)	4992.03	2	2496.01	42.63	4.42 × 10^−9^	3.35
MK contain × NaOH	470.37	4	117.59	2.01	1.22 × 10^−1^	2.73
Error	1581.03	27	58.56			
Total	64,470.56	35				

**Table 5:** Two-way ANOVA of the length change ratio at 28 days.

Source of Variations	Sum of Squares	DoF	Mean Square	F-Value	*p*-Value	Critical Value
MK Contain (%)	0.3812	2	0.1906	178.52	1.35 × 10^−12^	3.555
NaOH (M)	0.0126	2	0.0063	5.90	1.07 × 10^−2^	3.555
MK contain × NaOH	0.0332	4	0.0083	7.78	7.99 × 10^−4^	2.928
Error	0.0192	18	0.0011			
Total	0.4460	26				

**Table 6:** One-way ANOVA of the length change ratio at 28 days.

Source of Variation		Sum of Squares	DoF	Mean Square	F-Value	*p*-Value
NaOH concentration	MK0	0.0034	2	0.0034	19.74	2.29 × 10^−3^
	MK50	0.0188	2	0.0094	3.23	1.12 × 10^−1^
	MK100	0.0202	2	0.0101	85.16	3.94 × 10^−5^
MK content	6 M	0.2077	2	0.1039	449.09	2.92 × 10^−7^
	10 M	0.1244	2	0.0622	113.34	1.71 × 10^−5^
	14 M	0.0823	2	0.0412	16.99	3.38 × 10^−3^

**Table 7:** The coefficients β for the compressive strength and length change ratio at 28 days.

	Compressive Strength	Length Change Ratio
β_0_	21.2	4.25 × 10^−1^
β_1_	−1.74	−8.88 × 10^−4^
β_2_	16.28	1.97 × 10^−2^
β_3_	1.01 × 10^−2^	4.02 × 10^−5^
β_4_	−5.85 × 10^−1^	−1.25 × 10^−3^
β_5_	−2.23 × 10^−2^	2.22 × 10^−4^

**Table 8:** Comparison of the test result and prediction result.

Test	Prediction	Experiment	Error
Compressive strength at 28 days	32.8 MPa	34.9 MPa	6.04%
Shrinkage at 28 days	0.289%	0.287%	0.77%

## Data Availability

All data are contained within the article.
